# Perceived barriers and facilitators in providing palliative care for people with severe dementia: the healthcare professionals’ experiences

**DOI:** 10.1186/s12913-018-3515-x

**Published:** 2018-09-12

**Authors:** May Helen Midtbust, Rigmor Einang Alnes, Eva Gjengedal, Else Lykkeslet

**Affiliations:** 1 Faculty of Medicine and Health Sciences, Department for Health Sciences in Aalesund, Norwegian University of Science and Technology, Box 1517, NO 6025 Aalesund, Norway; 2 University of Bergen, Global Public Health and Primary Care, Box 7804, 5020 Bergen, Norway; 3 Faculty of Health Sciences and Social Care, Molde University College, Box 2110, NO 6402 Molde, Norway

**Keywords:** Palliative care, Dementia, Long-term care facilities, Qualitative approaches, Focus groups, Barriers, Facilitators

## Abstract

**Background:**

Dementia has become a major public health issue worldwide due to its rapidly increasing prevalence and an increasing number of dementia-related deaths in long-term care facilities. The aim of this study was to examine health professionals’ experiences of potential barriers and facilitators in providing palliative care for people with severe dementia in long-term care facilities.

**Methods:**

This was a qualitative descriptive study. The data were collected from four focus groups and 20 individual in-depth interviews with healthcare professionals from four Norwegian nursing homes. The data were analysed by thematic text analysis, as described by Braun and Clarke.

**Results:**

The major findings indicate that healthcare professionals experience a lack of continuity as the main barrier to facilitating palliative care. Time pressure and increased efficiency requirements especially affect the weakest and bedridden residents with dementia. The healthcare professionals feel conflicted between wanting to spend more time caring for each individual resident and feeling pressure to help everyone. Although resources are scarce, dying residents are always given priority by healthcare professionals, either by the hiring of extra personnel or the reorganization of tasks in a way that facilitates someone staying with the terminal resident. Advanced care planning was highlighted as a facilitator in providing palliative care, but the extensive use of temporary staff among nurses and doctors and the relocation between the sheltered and long-term wards threaten the continuity in planning and providing palliative care.

**Conclusions:**

The findings indicate that healthcare professionals experienced several structural barriers that prevented the provision of palliative care to people with severe dementia in long-term care facilities. Increasing demands for economic rationality lead to a lack of continuity of care. Organizational changes, such as measures to increase the competence and the proportion of permanent employees and the prevention of burdensome end-of-life transitions, should be implemented to improve continuity and quality of care.

**Electronic supplementary material:**

The online version of this article (10.1186/s12913-018-3515-x) contains supplementary material, which is available to authorized users.

## Background

Dementia has become a major public health issue worldwide due to its rapidly increasing prevalence. This increase poses challenges for providing proper care, including end-of-life care [1, 2]. Studies of the location of death for older people with dementia show that an increasing number of dementia-related deaths in the United States and Europe occur in long-term care facilities [3, 4]. Given the increasing prevalence of people living and dying with dementia in long-term care facilities, it is important to know more about health professionals’ perceived barriers and facilitators in providing palliative care.

Healthcare professionals’ perceived barriers and facilitators are affected by changes in the healthcare system. In the last few decades, the healthcare systems in developed countries have changed in the direction of New Public Management (NPM) to meet the demands for improved effectiveness and quality [5–7]. The ideology of NPM is based on productivity, standardization and economic rationality as fundamental principles [6]. In the 1990s, the Norwegian healthcare sector became part of this international reform trend, which was expressed through reforms such as the Hospital Reform and the Coordination Reform [8, 9]. These reforms have given municipalities and long-term care facilities new and demanding tasks that were previously performed by hospitals. In Norway, the long-term care facilities sector is an integrated part of the extensive social welfare system. An essential characteristic of this system is that the healthcare services are provided, either free or at highly subsidized prices, to all citizens in need, irrespective of socioeconomic status [10]. The lowest government level, the municipalities, is responsible for financing and providing accommodation in long-term care facilities to ensure necessary and proper health and care services for the population [11]. To ensure the quality of services, the government also requires municipalities to establish systems and procedures that contribute to, among other factors, dignified end-of-life care in a safe environment [12]. Municipalities are responsible for providing the necessary allocations and funding to the long-term care facilities, and the head nurses are responsible for managing the assigned budget [10, 13]. The decentralization of new and demanding tasks combined with the rapidly growing number of people with dementia presents municipalities and long-term care facilities with major care-related and financial challenges [8, 14].

Residents dying of dementia have significant healthcare needs, and palliative care, with its focus on comfort and quality of life, should be made available to everyone [15–17]. Despite the availability of generic definitions of palliative care, it is still unclear precisely what palliative care in dementia entails. The European Association of Palliative Care (EAPC) published a white paper defining the principles of palliative care in dementia [18], which include continuous, proactive, person-centred care with timely recognition of the dying phase, while at the same time providing comfort and psychosocial and spiritual support and avoiding unnecessary burdensome treatments. The importance of the collaboration between healthcare professionals and family caregivers when end-of-life care decisions are made and of the education of healthcare professionals were also emphasized [18, 19]. The healthcare systems, as outlined above, may be seen as hindering the provision of palliative care in terms of the EAPC definition. Studies show that the introduction of new reforms in the public sector has put healthcare professionals into long-term care facilities in a new situation with increasing demands and limited resources [19–22].

Most residents in long-term care require extensive care and treatment, and several studies report healthcare professionals’ lack of confidence in their skills and their need for more training and knowledge regarding dementia, pain assessment and behaviour problem management [15, 23, 24]. Advanced care planning (ACP) is considered an important component for the facilitation of palliative care, and it is particularly relevant for people with dementia who lose the capacity to make decisions at the end of life [25–28]. Robinson et al. (2012) defines ACP as “a multi-stage process whereby a patient and their carers achieve a shared understanding of their goals and preferences for future care”. When people with dementia move to a long-term care facility, communication about ACP should be introduced while they are still able to communicate their wishes [26, 29]. Support for families from both nurses and doctors is important to help them in their role as proxy decision makers [18]. Despite the evidence of potential benefits, which include quality of dying and increased family satisfaction [28, 30], studies show that healthcare professionals struggle with implementing ACP in long-term care facilities [26, 31]. A better understanding of dementia and the concept of ACP is required for ACP to be a useful tool in facilitating palliative care [26].

Earlier studies note several barriers influenced by the healthcare system, but studies of healthcare professionals’ experiences, with particular focus on potential conditions that may facilitate and/or constrain the provision of palliative care in the long-term care facility context, are less available. Hence, the aim of this study was to explore healthcare professionals’ experiences of potential barriers and facilitators in providing palliative care for people with severe dementia in long-term care facilities.

## Methods

### Design

This study had a qualitative, descriptive design as we searched in-depth understanding of the healthcare professionals’ experiences. We primarily used focus groups to collect data because this method works particularly well in exploring perceptions, thinking and feelings about a specific topic. Focus groups often present a more natural environment than individual interviews because the group interaction helps individuals to express different experiences. We aimed to bring about variation in the group members’ experiences and allowed for contrasting opinions on the topic. We succeeded in creating a permissive environment that encouraged the participants to have a natural and easy conversation without much interference from the researcher [32]. The focus group data were supplemented with insights from 20 individual interviews from a previous study. In that study, we interviewed the same informants to gain insight into their experiences of palliative care for people with severe dementia in long-term care facilities. Although these interviews did not focus on barriers and facilitators, this information was provided, which we used as a supplement in the present study [33].

### Participants and recruitment

The management teams of four long-term care facilities in mid-Norway were asked to recruit healthcare professionals for the study. To ensure some variation in the selection, two long-term care facilities in a mid-sized city and two long-term care facilities in smaller municipalities were randomly selected. The sizes of the four long-term care facilities were relatively similar; they had 48 to 78 beds and different units, such as short- and long-term wards. Three of them also had sheltered wards for residents with a dementia diagnosis. The care in a sheltered unit is adapted to residents with dementia, and there are usually fewer residents in these units than in short- and long-term units. We included both licensed practical nurses and registered nurses. Although registered nurses have primary responsibility for providing palliative care in long-term care facilities, practical nurses have a key role in direct patient care. The manager of each unit gave all the healthcare professionals a description of the study and a letter containing the same information. This description included information about the purpose of the study, researcher credentials, and the fact that participation was voluntary and that they could withdraw at any time without consequences. Those who wanted to participate in the study could either directly contact the first author or the manager of the unit. Ten registered nurses and six licensed practical nurses, four from each long-term facility, participated in the focus groups. The same informants plus four others, for a total of 20, had previously participated in individual interviews. The participants, all women, had an average age of 44 years (range 31–61). They were employed in half-time to full-time positions and had three to 37 years (average 18) experience working with people with dementia.

### Data collection

The first author (MHM) conducted all the individual interviews and was the moderator at the focus groups. She is a nurse and has experience from the field of dementia care and qualitative research. Two of the other authors (REA, EL) co-moderated three and one of the groups each. All the co-authors are nurses and are trained in qualitative research. Four focus groups, one at each location, and individual in-depth interviews with the healthcare professionals from the four facilities, were conducted. A semi-structured interview guide was used to balance openness and focus during the interviews. We arranged four focus groups because four long-term care facilities were included in the study. In addition, we then had the possibility to compare and contrast data across the groups. The focus groups were scheduled for four to five participants based on recommendations to use smaller groups when the participants have substantial experiences with the topic [32]. The moderator opened the group interviews with the following question: What are your experiences with facilitating palliative care for people with severe dementia in long-term care facilities? Follow-up questions regarding potential barriers and facilitators were asked during the interview (for complete interview guide, see Additional file 1). We emphasized that the participants had enough time to respond, but the group members quickly established a dialogue, and the conversation flowed easily. The moderator interrupted only when she wanted to move the conversation to new topics. At the end of the interview, the co-moderator summarized the main points and gave the participants an opportunity to further comment on the different topics. The individual interviews started with a similar opening question: What are your experiences with providing palliative care for people with severe dementia in long-term care facilities? (for complete interview guide, se Additional file 2). All the interviews were conducted in suitable meeting rooms at the current location. Focus groups were conducted during the time overlap between the day and afternoon shifts and lasted for approximately 90 min. The individual interviews were conducted during the day shift and lasted from 45 to 60 min. All interviews were tape-recorded and transcribed verbatim by the first author. All places, names and identifiable information were anonymized under transcription.

### Data analysis

The two data sets were first analysed separately. Both the transcripts from the focus group and the individual interviews were analysed using a method inspired by thematic text analysis, as described by Braun and Clarke [34]. This analysis was divided into several steps. First, the interview transcripts were read several times to allow the readers to become acquainted with the content. The data were then organized by coding or dividing the text into meaningful elements. The coding process continued until all the data were exhausted. NVivo 11 qualitative data analysis software was used for the coding of the text. The next step was to identify themes by grouping related codes. Preliminary themes from both data sets were then discussed and adjusted until the research group agreed on some final themes that illuminated the purpose of the study. All the authors read the main impressions of each interview as a group. They participated in the stepwise analysis and discussions along the way. An illustration of the analysis process is presented in Table 1.

**Table 1:** Illustration of the analytical steps

Quotes	Coding	Preliminary themes	Subthemes	Major theme
“We have a hectic department now, and we have had it for a long time. In addition, with two bedridden patients with severe dementia approaching [in] the last stage of life, we do not have time to be with them as much as we want.” (Focus group two)	Lack of resources prevent healthcare professionals from facilitating good palliative care	Time pressure	Lack of resources – a dilemma	Threats to continuity
“We are the few staff at work, but we prioritize patients in the terminal phase. However, others may suffer a little and get less attention.” (Focus group three)	Lack of resources means that healthcare professionals have to make tough choices	Prioritizing one patient means that other patients get less attention	Lack of resources – a dilemma	Threats to continuity
“We get sicker and sicker patients with more demanding diagnoses, but the resources do not increase.” (Practical nurse, individual interview)	The resources do not increase as the demands increase	Too little time and too few resources	Lack of resources – a dilemma	Threats to continuity

## Results

### Threats to continuity

The main finding of this study was that healthcare professionals experienced the lack of continuity as a major threat to the palliative care of people with severe dementia in long-term care facilities. Time pressure and an increasing demand to be efficient had an especially strong effect on the weakest residents with dementia. The healthcare professionals felt the pressure of having a guilty conscience for spending too little time with each resident in conflict with the pressure of getting around and helping everyone. The scarcity of resources meant that the professionals were unable to provide the care they wanted to until the resident with dementia was at the very end of life. At that point, the residents are always given priority by either hiring extra personnel or by the professionals reorganizing their tasks in a way that facilitates someone staying with the terminal resident. Advanced care planning was highlighted as a facilitator for providing palliative care, but the extensive use of temporary staff among nurses and doctors and the relocation between the sheltered and long-term wards threaten the continuity in planning and providing palliative care.

The threat to continuity is described in the following subthemes: lack of resources – a dilemma, end-of-life transitions – a paradox, and care planning – ideals versus realities.

### Lack of resources – A dilemma

Several of the participants underlined time pressure and a working culture marked by business and tough priorities. Changes in the healthcare service have led to new and demanding tasks. The healthcare professionals noted that the residents in long-term care facilities are increasingly sick and need increasing amounts of help. However, resources do not increase as tasks progress, and although the professionals are required to perform new and more demanding tasks, the leaders of long-term care facilities order them to follow economic measures. The healthcare professionals in long-term wards in particular reported experiencing pressure over time, which impacts the weakest residents and bedridden residents with a severe degree of dementia. These professionals felt inadequately able to be present for the weakest residents while at the same time getting around and helping all other residents. People with dementia become a vulnerable group with a large degree of uneasiness when no one has time to sit down with them. When one resident expresses unrest, the disturbance spreads throughout the whole ward. Too few healthcare professionals working in a ward with residents with various degrees of dementia and other residents who demand extensive physical care led to physical and mental exhaustion among the professionals. One nurse said that the time pressure makes her feel guilty towards the residents who often express the desire for her to sit down with them, but she always feels the need to rush along to perform her other responsibilities as a nurse. She told a story about an old woman with dementia. The woman was uneasy and subject to hallucinations, but she calmed down when the staff sang well-known songs for her. The nurse was frustrated that she and other staff often do not have time to provide such care for the residents: “*We work like robots….we need more time with the residents”*.

One of the licensed practical nurses said that palliative care for residents with severe dementia demands knowledge and precise observations. When she has little time and is stressed by all the tasks she must perform, it is difficult to interpret each resident’s expressions, which hinders her ability to provide good care. Several of the participants underlined that security and calmness is necessary for residents with dementia, but this can be difficult to provide in a ward where staff must run back and forth constantly. One licensed practical nurse said, “*It is not knowledge but resources that hinder good palliative care*”. The same nurse said she also wants resources to provide good palliative care before the terminal phase. The staff observe residents approaching the end of life, but the scarcity of resources denies them the ability to provide the care they want to offer before the resident reaches the very end of life.

At that point, the resident is given priority, and extra personnel are hired if needed so that someone can be with the terminal resident at all times. If the management informs them that there are not enough resources to hire extra personnel, the healthcare personnel are loyal to the system and run even faster to ensure that someone can be with the resident and the relatives. The group had the following dialogue about terminal care:

*Nurse 1) We are few, the resources are scarce, but we give priority to the residents who need it. This at the expense of the others, who receive less attention. If someone demands that we be there all day, we are.*

*Nurse 2) Yes, and we get coverage for night shifts, regular shifts at night.*

They try to arrange for a nurse or a licensed practical nurse who knows the terminal resident to stay with him or her; however, this requires experienced and competent healthcare personnel to provide care on the ward. If only new personnel are left on the ward, repeated requests disturb the ward and the dying resident. Some relatives are present day and night during a resident’s last days, and health personnel stated that despite scarce resources, they try to take care of both the resident and the relatives in the best way possible. They offer food and drinks and provide beds if the relatives want to stay overnight. If the resident had no relatives or his or her relatives are rarely present, the professionals reorganize their tasks in a way that opens up time for someone to stay with the terminal resident. One nurse said, “*It is a golden rule, a long-term care facility rule that nobody shall die alon*e”.

The healthcare personnel noted that providing attendance and close follow-up during the terminal phase is critical for allowing the resident and his/her relatives to feel safe. Good palliative care also demands personnel who have extensive knowledge of the resident and the competence necessary to provide both medical and non-medical treatment at the appropriate starting point for each resident.

### End-of-life transitions – A paradox

Three out of the four focus groups included participants who worked on sheltered wards for residents with dementia. One of the topics emphasized by the participants in this group as an obstacle to providing good palliative care was relocation among different wards. When a resident with dementia is moved, it is mostly because of a loss of bodily functions and a progression of their disease. They need more extensive care, and when they no longer function at a certain level, they are moved to the sheltered ward. The health personnel said that different criteria can be used as reasons for relocating a resident and that most often, residents are moved when they can no longer walk on their own and have to remain in a wheelchair or in bed. When residents are moved, they lose access to the health personnel who have come to know the resident and his or her relatives over the years. The paradox is that residents are moved when they are at the weakest and have the greatest need for personnel who can interpret and understand their individual expressions. Important information is often lost when residents are relocated, and professionals have to start anew with residents who are so severely ill with dementia that they no longer can express their wants and needs. A dialogue in the group illustrated the frustration felt among health personnel:

*Nurse 1: And I think a lot of information disappears when a resident moves from one ward to another.*

*Nurse 2: Yes, and we have to start all over again to build trust.*

*Nurse 1: All over again, that is true; you find some information in the resident’s report, but the small things?*

*Nurse 2: Yes, the details.*

*Nurse 1: Details that are of major importance.*

The healthcare professionals felt that it was very demanding to relocate and receive the most seriously ill residents at a phase in which they need familiar nurses around. They all agreed that moving among wards is particularly damaging for residents with dementia and that the residents often enter a phase of deterioration in connection with being relocated.

The relatives are often also affected when their family member is relocated, and healthcare professionals are often told by relatives that the resident was better in a safe and known environment. A licensed practical nurse said that the relatives depend on the help and care their family member receives. She said that relatives suffer something similar to a grief reaction when the resident has to move. Both the resident and the relatives must adapt to new routines, not the least being new and unfamiliar personnel who know neither them nor their family member. Long-term wards in many nursing homes are organized with long corridors and with numerous residents and healthcare personnel moving from room to room. In the sheltered wards, the atmosphere is quieter and specifically adapted to residents with dementia. Residents who come from a sheltered unit are used to having the personnel around in the living room, whereas on the other wards, the personnel are more occupied with nursing and providing care in the resident’s room. Healthcare professionals have less time to spend with residents in common areas, and this leads to uneasiness and insecurity among the residents with dementia. A licensed practical nurse said that it is so sad to move residents from sheltered to long-term wards since long-term units are not organized for residents with a severe degree of dementia. “*Sadly, this is the system*”, said a registered nurse, expressing a feeling of powerlessness towards the system.

### Care planning – Ideals versus realities

Care planning requires cooperation among relatives, nurses and doctors, but the healthcare professionals considered the extensive use of temporary help among nursing staff and doctors an obstacle to care planning and providing palliative care. The use of temporary staff threatens the continuity and knowledge of the group of healthcare professionals. Most of the hired help are unskilled, and they form a large group within the staff of long-term care facilities. Some temporary staff members from agencies are registered nurses and have the necessary skills, but their lack of knowledge over time and their lack of competence in interpreting each resident’s body language complicate the planning and facilitation of good palliative care among residents with a severe degree of dementia. Another challenge emphasized was the lack of knowledge of Norwegian among nurses recruited from agencies. A licensed practical nurse said the following: “*As long as you have the right title, it is fine, but it can be very frustrating when the resident perhaps has lost parts of his language and has hearing loss as well, when the nurse caring for him is incapable of conveying the language… It is not easy…. And people with dementia are suffering the most”.*

A familiar long-term care facility doctor is also a decisive factor in planning and providing palliative care. Several nurses emphasized that they felt safe when they had a familiar long-term care facility doctor who makes decisions based on assessments performed over time, in cooperation with the healthcare professionals and the resident’s relatives. In the healthcare professionals’ experience, the regular long-term care facility doctor wants to plan for the resident’s future. A nurse said that she discusses ethical problems concerning residents’ medical care on every doctor’s round. Although the healthcare personnel at two out of four long-term care facilities had the positive experience of having a regular long-term care facility doctor, everyone had experiences with temporary doctors at some point. In one of the long-term care facilities in particular, the healthcare professionals found it very demanding not to have regular doctors. They said that over a period of a few years, they had as many as 13 different long-term care facility doctors, and one nurse said, “*The temporary doctors mainly conduct damage control and lack the continuity that is important to both the resident and the relatives”.*

The healthcare professionals described how they, in cooperation with the doctor and the residents’ relatives, make plans for how residents with dementia can end their life in the best possible way. At one of the long-term care facilities, the use of advanced care planning (ACP) was introduced a few years ago. ACP is a structured plan to ensure cooperation, planning and facilitation of palliative care. The participants described ACP as a well-structured plan that allows for questions about resuscitation, hospital admissions, treatment of infections, intravenous treatment, and medical and non-medical treatment to be discussed among doctors, nurses, relatives and, if possible, the resident. The plan facilitate continuity and secure the care of each resident, regardless of which professionals are at work. ACP is especially a facilitator for providing palliative care for residents with severe dementia because they are unable to express their wishes and needs towards the end. Functioning cooperation with the relatives is, in many ways, the voice of the resident and is of great value in preparation of the plan. The relatives participate along with the healthcare professionals and the doctor in decisions concerning the end of life. One nurse said about the palliative plan, “*This is a very good working tool. One can ask the tough and difficult questions and write them down for everyone involved”.*

Although only one of the long-term care facilities actively used a standardized ACP, the healthcare professionals at the other long-term care facilities had experience with a broader focus on planning for palliative care through meetings with relatives. These meetings are used as an arena for touching on upon treatment intensity and providing information on the resident’s health condition. The participants said that meetings with the relatives were to be arranged at least once a year and more often if there were changes in the resident’s health conditions. Both regular meetings with relatives and the use of ACP are emphasized as a facilitator for providing palliative care, whereas the extended use of temporary doctors and nurses threatens the continuity of both planning and providing palliative care.

## Discussion

The purpose of this study was to explore healthcare professionals’ experiences of potential barriers and facilitators in providing palliative care for people with severe dementia in long-term care facilities. The major findings indicate that healthcare professionals experience a lack of continuity as the main threat to facilitating palliative care. Time pressure and increased efficiency requirements most strongly affect the weakest and bedridden residents with dementia. Healthcare professionals feel conflicted between wanting to spend more time caring for each individual resident and being able to help everyone. Despite scarce resources, dying residents are always given priority by either the hiring of extra personnel or the professionals’ reorganization of their tasks in a way that facilitates someone staying with the terminal resident. Advanced care planning (ACP) was experienced as a facilitator for planning and providing palliative care, but extensive use of temporary staff among nurses and doctors and resident relocation from sheltered to long-term wards threaten continuity in planning and providing palliative care.

Given the growing prevalence of people living and dying with dementia in long-term care facilities [2–4], it is important to understand more about healthcare professionals’ conditions for providing palliative care. The findings of this and other studies show that the introduction of New Public Management (NPM)-inspired reforms has created a new situation for healthcare professionals in long-term care facilities with increasing governmental demands and limited resources [19–22]. The economic rationalism operating in long-term care facilities is influenced by NPM, the governmental political ideology. With such an ideology, there may be a risk that the number of treated patients is more heavily weighted than the quality of care provided [35]. Professionals feel conflicted between their ever-increasing demands and responsibility to facilitate good palliative care for the individual. Previous studies have shown that increasing demands for a cost-effective healthcare system have resulted in an exacerbation of conflicting values that make it increasingly difficult for healthcare professionals to balance such tensions [36, 37]. One consequence of not being able to effectively negotiate these interception values has been identified as moral distress [36]. In its original form, moral distress was defined by the philosopher Andrew Jameton as when “one knows the right thing to do, but institutional constraints make it nearly impossible to pursue the right course of action” [38]. The term has been further developed by researchers, and moral distress is now recognized as a phenomenon that affects healthcare professionals in different arenas of practice and as a phenomenon that reflects providers’ difficulties fulfilling their moral responsibility [36].

The findings of this study indicate that increasing demands for economic rationality, which prevails in the NPM ideology [6], prevent the facilitation of good palliative care for residents with dementia in long-term care facilities. The informants in this and other studies experienced that patients who come to long-term care facilities are increasingly sick and need increasing amounts of help [20, 21]. Despite the introduction of new and demanding tasks, resources are not increased accordingly, and the leaders of long-term care facilities order healthcare professionals to save even more costs to adhere to the assigned budget. The healthcare professionals felt the pressure of having a guilty conscience for spending too little time with each resident in conflict with the pressure of getting around and helping everyone. They felt conflicted by the demands for efficiency and their moral responsibility during face-to-face meetings with residents and relatives. The scarcity of resources denies them the ability to provide the care they want to provide before the resident reaches the very end of life. At that point, the residents are always given priority through either the hiring of extra personnel or through the healthcare professionals’ reorganization of their tasks in a way that facilitates someone staying with the terminal resident.

The economic rationale also manifests in the extensive use of temporary staff. The findings in this study show that a high proportion of temporary staff creates challenges among healthcare professionals in terms of both competence and continuity. Due to the cost-effective nature of long-term care facilities, the use of temporary staff seems to increase. Calculations show that approximately one-third of the personnel in Norwegian long-term care facilities are without health or social training [39, 40]. Previous studies note that the healthcare sector’s agenda to reduce costs results in poor staffing levels, high staff turnover, demanding workloads, low pay and low job satisfaction, resulting in high levels of role burden [19, 22]. The efficient healthcare system constrains healthcare professionals from providing good palliative care, and they are prone to moral stress because they are not able to fulfil their moral responsibility. Employing low-paid staff with minimal skills and training has previously been shown to have a detrimental impact on the provision and facilitation of palliative care for people with dementia [19, 24]. Temporary staff do not have the long-term familiarity and knowledge of how to “read” and observe a patient with severe dementia [33] and thus cannot provide continuous person-centred palliative care [18]. The need for increased competence has been noted in previous studies [15, 23, 24], and it was a key goal of the Norwegian Dementia Plan 2015. Educational tools such as Dementia ABC have been developed to provide necessary and up-to-date information on dementia and environmental treatment for all occupational groups working with people with dementia. Dementia ABC has been used in 94% of the Norwegian municipalities, but only a minority of nursing staff have followed these programmes much because they have not been made available to temporary staff or part-time workers [40].

The healthcare professionals in this study also experienced being confronted with moral distress when the weakest residents were moved from safe and familiar surroundings in the sheltered ward to a long-term ward. Previous research regarding end-of-life transitions among long-term care facilities residents with dementia shows that transitions between different types of residential care facilities and hospitals are common and present major challenges to the continuity of end-of-life care [41–43]. The paradox is that the patients are moved when they have entered a phase of the disease when they most need familiar healthcare professionals who understand their manners of expression [33]. The use of such a system of end-of-life transitions may be viewed as a consequence of ongoing changes in the healthcare sector, materialized as expectations of economic rationality and standardization of practice [6]. Residents with dementia are moved from a sheltered ward specially organized for residents with dementia (economic rationality) when they no longer are able to walk on their own (standardization). More standardization of practice weakens clinical autonomy [44], and the informants in this study felt powerless against a system in which their knowledge and assessments of what is best for the resident were overridden.

ACP may also be viewed as an example of the standardization of practice, but unlike end-of-life transitions, the use of a standardized ACP was highlighted as a facilitator of planning and providing palliative care for residents with severe dementia. ACP is important for a shared understanding of the goals and preferences for future care [31]. Nevertheless, studies show that healthcare professionals struggle with ACP implementation in long-term care facilities [26, 31]. Although ACP was perceived as a good working tool for facilitating palliative care, the informants experienced the use of temporary workers, both nurses and doctors, as a main barrier for planning palliative care. Regular meetings with relatives, nurses and doctors are a significant component in the planning of palliative care, and the availability of regular doctors is a prerequisite for the existence of such meetings. The lack of availability of doctors was also confirmed as a barrier to facilitating palliative care in earlier research [45].The healthcare professionals in our study emphasized that having a familiar long-term care facility doctor who knows the resident and his or her family well is crucial for planning and providing palliative care.

The findings of this study show that healthcare professionals are exposed to increasing demands for economic rationality and standardization of practice combined with the moral responsibility to provide good palliative care for residents with severe dementia. Healthcare professionals experienced that structural barriers led to a lack of continuity of care, which they considered to be a main threat to facilitating palliative care.

### Strengths and limitations

We consider it a strength that we gained information from healthcare professionals with different levels of education working in different units: short- and long-term wards and sheltered wards for people with dementia at four different long-term care facilities. When healthcare professionals from different units were put together in focus groups, we gained insight into the experiences of informants who worked under dissimilar framework conditions and knowledge about how these experiences affected the facilitation of palliative care for people with severe dementia. It is also a strength that the findings from the focus groups are consistent with the findings from the individual interviews. In addition, we believe our close cooperation and reflections in the research group through all stages of the research process are a strength in this study.

The study has its limitations; in particular, part of the recruitment of the informants may have had some deficiencies. Some of the participants contacted the first author directly, but approximately half of the informants were recruited through the manager of the unit. Recruitment may have been influenced by the preferences of the management; they may have chosen informants that they thought were suitable, and other potential informants who might have added important information may have been excluded. Although managers should have been selective in recruitment, it does not seem that this practice has prevented participants from daring to be critical of the health system.

## Conclusion

This study presents healthcare professionals’ experiences of barriers and facilitators related to the facilitation of palliative care for people with severe dementia in Norwegian long-term care facilities. The EAPC definition of palliative care in dementia emphasizes the importance of continuous, holistic and person-centred care [18]. The findings of this study, however, indicate that healthcare professionals experience several structural barriers that complicate the facilitation of palliative care according to the EAPC definition. Increasing demands for economic rationality and standardization of practice lead to a lack of continuity in care, and the healthcare professionals noted this as the main threat to facilitating palliative care. Although resources are scarce, dying residents are always given priority by healthcare professionals either by the hiring of extra personnel or by the reorganization of tasks in a way that facilitates someone staying with the terminal resident. Advanced care planning was experienced as a facilitator for providing palliative care, but extensive use of temporary staff and patient relocations between sheltered and long-term wards threaten the continuity of planning and providing palliative care. The findings suggest a need for organizational changes with a greater focus on quality of care. Quality as well as economic rationalism is a goal in the NPM ideology.

The findings of this study contribute to an understanding of how healthcare professionals’ barriers and facilitators affect the facilitation of palliative care for people with severe dementia in long-term care facilities. For healthcare professionals to be able to facilitate palliative care for the rapidly increasing population living and dying with dementia in long-term care facilities, organizational changes are needed. At present, long-term care facilities are not sufficiently adapted to the needs of people with dementia and their families. Measures to increase competence and the proportion of permanent employees and to prevent burdensome end-of-life transitions should be implemented to improve continuity and quality of care.

## Additional files

Additional file 1: Interview guide focus groups. (DOCX 13 kb)

Additional file 2: Interview guide individual interviews. (DOCX 13 kb)

## Abbreviations

ACP: Advanced care planning.
EAPC: The European Association of Palliative Care
NPM: New Public Management
