# Supplementary material for: Perceived barriers and facilitators in providing palliative care for people with severe dementia: the healthcare professionals’ experiences
Source: BMC Health Serv Res. 2018 Sep 12;18:709. doi: 10.1186/s12913-018-3515-x (PMC6134769; doi:10.1186/s12913-018-3515-x)
Supplement: Supplementary file 1 — Interview guide focus groups. (DOCX 13 kb) [file 12913_2018_3515_MOESM1_ESM.docx]

# Interview guide focus groups

1. **What are your experiences with facilitating palliative care for people with severe dementia in long-term care facilities?**
2. Free discussion
3. Follow-up questions regarding:
4. What are your experiences regarding potential barriers in providing palliative care for people with severe dementia in long-term care facilities?
5. What are your experiences regarding potential facilitators in providing palliative care for people with severe dementia in long-term care facilities?
