# Supplementary material for: Perceived barriers and facilitators in providing palliative care for people with severe dementia: the healthcare professionals’ experiences
Source: BMC Health Serv Res. 2018 Sep 12;18:709. doi: 10.1186/s12913-018-3515-x (PMC6134769; doi:10.1186/s12913-018-3515-x)
Supplement: Supplementary file 2 — Interview guide individual interviews. (DOCX 13 kb) [file 12913_2018_3515_MOESM2_ESM.docx]

# Interview guide individual interviews

1. **What are your experiences with providing palliative care for people with severe dementia in long-term care facilities?**
2. **What do you associate with good palliative care for people with severe dementia in long-term care facilities?**
   1. Please give examples.
3. **Do you have experiences with situations where you did not succeed in providing good palliative care for people with severe dementia in long-term care facilities?**
   1. Please give examples.
4. **What significance has providing palliative care for people with severe dementia in long-term care facilities had for you?**
   1. Relationship to the person with dementia.
   2. Relationship to family caregivers.
5. **What do you think is most challenging in providing palliative care for people with severe dementia in long-term care facilities?**
   1. Please give examples.
